# Beyond Bulk Metabolomics: Emerging Technologies for Defining Cell-Type Specific Metabolic Pathways in Health and Disease

**DOI:** 10.3390/biom15121687

**Published:** 2025-12-02

**Authors:** Yichen Gong, Samuel Weinberg

**Affiliations:** 1 Department of Pathology, Northwestern University Feinberg School of Medicine, Chicago, IL 60611, USA; yichengong2028@u.northwestern.edu; 2 Center for Human Immunobiology, Northwestern University Feinberg School of Medicine, Chicago, IL 60611, USA

**Keywords:** immunometabolism, rare immune cell metabolomics, single-cell metabolic profiling, LC-MS HILIC, CRISPR screening

## Abstract

While metabolomics has emerged as a powerful tool for discovering disease biomarkers, the clinical utility of plasma or tissue metabolite profiles remains limited due to metabolic heterogeneity and flexibility across cell types. Traditional bulk metabolomics fails to capture the distinct metabolic programs operating within rare cell populations that often drive disease pathogenesis. This review examines cutting-edge approaches that overcome these limitations by characterizing metabolism at single-cell and cell-type-specific resolution, with particular emphasis on rare immune cell populations as a proof of concept. We discuss how the integration of flow cytometric metabolic profiling, molecular techniques, advanced metabolomics platforms, and computational modeling enables unprecedented insight into cell-intrinsic metabolic states within physiological contexts. We critically evaluate how these technologies reveal metabolic plasticity that confounds bulk measurements while identifying cell-type-specific metabolic vulnerabilities. Finally, we address the crucial challenge of establishing causality in metabolic pathways, a prerequisite for translating metabolomic discoveries into clinically actionable interventions. By moving beyond descriptive metabolomics toward a mechanistic understanding of cell-type-specific metabolism, these approaches promise to deliver the precision required for effective metabolic targeting in disease.

## 1. Introduction

Cellular metabolism has historically been viewed as acting secondary to nuclear instruction, primarily providing the bioenergetic and biosynthetic support necessary for cellular function [1]. This paradigm has been challenged over the last 20 years, and it is now clear that cellular metabolism plays a central signaling role and, in many cases, actually dictates cellular fate and function [2]. Beyond generating ATP and building blocks for macromolecular synthesis, metabolic intermediates serve as direct signaling molecules that orchestrate cellular programs. For example, intermediates of the tricarboxylic acid (TCA) cycle can function as cofactors or inhibitors of epigenetic enzymes, with succinate inhibiting α-ketoglutarate-dependent dioxygenases and acetyl-CoA serving as a substrate for histone acetylation. Similarly, metabolic processes generate reactive oxygen species (ROS) that function as signaling molecules, modulating redox-sensitive transcription factors and protein function to influence cell fate decisions [3]. This recognition that metabolism functions as both a cellular fuel source and a sophisticated signaling network has fundamentally transformed our understanding of how cells integrate environmental cues to control their fate and function.

The immune system exemplifies how metabolic regulation governs cellular fate and function, with mounting evidence demonstrating that nutrient availability, oxygen tension, and metabolite concentrations in tissue microenvironments profoundly shape immune cell responses [4,5]. Cellular ATP levels can regulate the AMPK energy-sensing pathway, which controls immune cell differentiation and effector programs, while sustained metabolic stress can drive durable transcriptional and epigenetic changes that alter immune cell function. Metabolites, including lactate, kynurenine, and adenosine, can act as direct immunomodulators, influencing everything from T cell activation to regulatory T cell (Treg) function [6,7]. Despite this growing appreciation for the central role of immunometabolism in human health and disease, a major challenge remains in understanding how these metabolic regulatory mechanisms operate within rare immune cell subsets that are largely tissue-resident and present in extremely low numbers. These rare populations, which include macrophages [8], hepatic stellate cells (HSCs) [9], tissue-resident memory T cells [10], specialized dendritic cell subsets [11], and localized Treg populations [12], often exert disproportionate functional effects relative to their abundance, yet their metabolic regulation remains poorly understood due to technical limitations in studying their metabolism within physiologic contexts. To fill this critical knowledge gap, we need to develop and apply novel approaches capable of assessing metabolism in these rare but functionally important immune cell populations. In this review, we examine cutting-edge technologies and methodological advances that enable metabolic characterization of immune cell subsets within their native tissue environments, discuss the limitations of current approaches on rare cell populations, highlight emerging tools that promise to overcome these barriers, and outline future directions for precision immunometabolism (Table 1).

## 2. Transcriptomic-Based Approaches to Investigating Metabolism in Rare Immune Cells

Over the past decade, the emergence of single-cell RNA sequencing (scRNA-seq) technologies has fundamentally transformed our ability to study rare immune cell populations [13]. These approaches have provided unprecedented resolution into transcriptional and functional heterogeneity of previously inaccessible cell types within their native tissue environments. In the immune system, this has revealed the diversity and specialization of tissue-resident lymphocytes, dendritic cell subsets, and regulatory T cell populations that were largely invisible in bulk analyses. Despite these advances, transcriptomic data alone provides an indirect proxy for metabolic function. Cellular metabolism is governed not only by gene expression but also by enzyme kinetics, substrate availability, and post-translational regulation, all of which shape actual metabolic flux. To enhance the value of scRNA-seq data, researchers have increasingly sought to expand scRNA-seq-based analysis by developing computational frameworks that map transcriptomic data onto curated biochemical and metabolic network information. These approaches aim to infer metabolic pathway activity and flux at the single-cell level, providing new avenues to explore how metabolism governs the behavior of rare immune subsets in vivo.

Among these approaches, the integration of scRNA-seq data with genome-scale metabolic models (GEMs) has emerged as a powerful strategy to predict cell-intrinsic metabolic states. GEM databases record the biological functions of individual metabolic genes, providing a scaffold for simulating metabolic flux and pathway activity under defined constraints [37]. Commonly used GEMs include BiGG Models [38], BioModels [39], Human GEMs (Recon1 [40], Recon2 [41], Recon 3D [42]), and form the basis of the computational tools used to predict metabolic behavior from gene expression datasets. One widely used in silico approach is Compass, which provides metabolic information from scRNA sequencing datasets [14]. Compass integrates differential gene expression data with existing knowledge of enzyme kinetics and regulation to predict metabolic network activity by building a Flux Balance Analysis (FBA) model to predict cell metabolic state. Specifically, it generates a score for each metabolic reaction, reflecting how active each reaction is predicted to be based on scRNA-seq data input. By combining these individual scores within specific pathways, Compass can identify global metabolic shifts in rare cell populations. Wagner and colleagues demonstrated the value of this approach to identify novel metabolic drivers of Th17 cell functional variability [14]. Specifically, Compass identified a metabolic switch between glycolysis and fatty acid metabolism underlying Th17 cell pathogenicity [14]. Further, Compass also predicted that this metabolic profile results in increased polyamine metabolism, which was causally demonstrated to drive Th17 cell inflammatory activity. Despite the power of this computational approach, cell metabolism is not dictated solely by intrinsic programs, as cells continually sense and adapt to changing exogenous metabolite levels in physiological microenvironments [43,44], motivating the development of frameworks that formalize this extrinsic metabolic crosstalk.

Another recently developed model, MEBOCOST, aims to assess this crosstalk by utilizing scRNA-seq data to investigate metabolic signaling between 2 cells [15]. Specifically, this algorithm identifies cell pairs as a potential “sender” and “receiver” for a given metabolite by jointly evaluating the expression of metabolic enzymes in the sender cells and the expression of sensor genes in the receiver cells. Every single metabolite released is identified as a metabolite-mediated cell–cell communication (mCCC) event. Compared to classical ligand-receptor-based cell–cell communications (CCC), which considers only cell surface receptors, mCCC also incorporates cell surface transporters [45] and nuclear receptors [46]. This expansion better captures the complexity of metabolic signaling and enables more comprehensive mapping of metabolic networks. Importantly, this method has been applied to human adipose tissue in the context of obesity and identified distinct mCCC events in low-BMI and high-BMI individuals. Further, MEBOCOST analysis identified macrophages and endothelial cell metabolic crosstalk as potential contributors to obesity-specific mCCC alterations. In comparison, Compass identifies altered within-cell metabolic states, while MEBOCOST charts between-cell metabolite exchanges. Together, they give an integrated blueprint that links intracellular flux programs to intercellular metabolic crosstalk and generates testable pathway-level hypotheses.

While these computational frameworks have greatly expanded our capacity to infer metabolic states from transcriptional data, they also come with notable limitations. Most current algorithms rely on predefined metabolic networks and assume steady-state flux conditions that may not accurately capture the dynamic nature of metabolism in vivo. Moreover, their predictive power is constrained by the completeness and accuracy of existing GEMs, which are often derived from well-characterized cell types and may not fully represent the metabolic landscape of rare immune populations. To address these limitations, Model Extraction Methods (MEMs) have been developed to refine and tailor GEMs to specific biological contexts [47,48,49,50]. These approaches use training datasets that include both scRNA-seq data and annotated metabolic pathway data to improve predictions when building algorithms. This approach is particularly effective when the target cell type matches the cell type used in the training data. However, when applied to different cell types or physiologic contexts, variations in baseline metabolism can lead to reduced accuracy, limiting the model’s generalizability.

Taken together, computational transcriptomic approaches provide practical advantages for single-cell metabolism. They leverage the rapidly growing public scRNA-seq atlases (e.g., Tabula Sapiens/HCA) and datasets, enabling hypothesis generation and metabolic state analysis [51,52]. These tools deliver mechanistically interpretable, pathway-level metabolic readouts. However, the computational transcriptomic predictions are only as good as the GEM and flux balance parameter estimates. Wrong assumptions about metabolic pathways or medium availability could lead to predictions that are not biologically feasible. Further, metabolism is not only regulated at the RNA level, but it is also controlled post-transcriptionally, through protein stability, subcellular location, enzyme kinetic complexity, and substrate availability [53,54,55,56]. As a result, computational predictions based solely on transcriptomics data only reflect a possible metabolic state rather than the actual metabolic flux distributions in cells. This leaves a knowledge gap between RNA measurement and actual metabolic state, emphasizing the need for experimental validation to support these predictive models.

## 3. Approaches to Assaying Real-Time Metabolic Flux in Rare Cell Populations

Over the past two decades, techniques that directly measure mitochondrial and glycolytic activity in living cells have revolutionized metabolism research. These methods permit real-time assessment of key energetic parameters such as oxygen consumption, ATP production, and extracellular acidification [57,58]. For example, extracellular flux (XF) analyzers (e.g., Seahorse) monitor changes in extracellular oxygen and proton concentrations over time and convert these to cellular oxygen consumption rate (OCR) and extracellular acidification rate (ECAR) to capture the instantaneous metabolic activity of a cell population [59]. These assays revolutionized metabolic research by enabling kinetic, noninvasive measurements in live cells, which allows investigators to interrogate basal and maximal mitochondrial respiration [18,60], ATP mitochondrial production [61,62], proton leak [63], and glycolytic activity [57] in response to perturbations.

However, a significant constraint of these population-scale methods is their requirement for large cell numbers to generate reliable, reproducible signals. In conventional formats, Seahorse assays often require tens of thousands of cells, and 3–6 technical replicates are recommended for consistent and reproducible data [18]. The Seahorse XF HS Mini Analyzer is designed for limited cell numbers and has lowered the requirements down to 10^4^ to 10^5^ range, which allowed for the assessment of rare immune populations [18]. In recent years, this instrument has been used to study a variety of rare cell populations, including viral-specific CD8+ T cells [64], human fibroblasts [65], and rare tumor cell subsets [66]. However, despite these advances, Seahorse assays remain technically challenging for rare immune populations. The assay requires cells to adhere uniformly to a microplate surface and remain metabolically stable throughout the measurement, which can be challenging for cells that are sensitive to local environmental inputs that cannot be recreated during the assay. Recent computational advances have allowed metabolic analysis generated from Seahorse assays to be combined with scRNA-seq datasets to better understand how individual cells utilize metabolism. For example, single-cell Flux Balance Analysis (scFBA) [19], which combines Seahorse-derived mitochondrial and glycolytic activity parameters with scRNA-seq profiles. By using both types of information, scFBA estimates how different pathways are working in each cell, creating a map of predicted metabolic activity. This approach is attractive because it links population-level metabolic measurements with single-cell gene expression data, which takes advantage of both high-level metabolism and cell-to-cell differences. Together, this platform provides a more complete and physiologically relevant picture of cellular metabolism.

A major downside of Seahorse assays is that they only report population-level metabolism rather than single-cell readouts. To address this constraint, several groups have developed flow cytometry-based approaches to obtain analogous metabolic information at single-cell resolution. The first of these, SCENITH (Single-cell energetic metabolism by profiling translation inhibition), works by perturbing major energy-generating pathways (e.g., glycolysis or mitochondrial respiration) with pharmacologic inhibitors and then quantifying the consequent change in ribosomal activity by pulsing puromycin and measuring its incorporation by antibody staining [27]. The central assumption of this method is that when ATP supply is limited, translational output falls. Thus, the larger the puromycin signal drop under a given inhibition, the greater the cellular dependence on that pathway for ATP generation. In contrast to Seahorse, SCENITH infers pathway reliance indirectly through translation, enabling single-cell multiparameter profiling in heterogeneous or rare immune populations. CENCAT (cellular energetics through noncanonical amino acid tagging) is analogous to SCENITH but uses ncAA incorporation and click labeling instead of puromycin staining, thus reducing toxicity [28]. Importantly, since SCENITH and CENCAT infer pathway dependence from changes in protein synthesis rather than direct fluxes, their readouts can be confounded by non-metabolic regulators of translation, such as stress responses or activation-dependent signaling. Thus, alongside matched controls or complementary flux measurements are needed for comprehensive interpretation. Other flow cytometry-based methods measure specific metabolic pathways by quantifying the levels of specific metabolic enzymes. Ahl et al. developed Met-Flow, a 27-parameter panel including 10 rate-limiting enzymes/transporters and phenotypic markers for 11 main leukocyte subsets [29]. This initial approach was further enhanced by applying spectral flow cytometry to increase the readout accuracy [67]. Importantly, a recent study combined a Met-Flow-like spectral flow cytometry-based panel with SCENITH to characterize T cell energetic metabolism, allowing for a combined flow cytometry-based single-cell metabolism measurement platform [68]. Together, these cytometry-based strategies offer powerful, single-cell, multiparameter readouts of metabolic dependencies across heterogeneous immune subsets and even limited clinical samples. At the same time, they infer metabolism indirectly via translation or protein abundance and therefore can be confounded by non-metabolic regulators of protein synthesis or activation-induced signaling. Thus, key findings should be validated with orthogonal flux-level assays.

Both Seahorse and flow cytometry–based metabolic profiling approaches bring important complementary strengths. Bulk assays like Seahorse provide real-time, functional, kinetic measurements (e.g., oxygen consumption, acidification) that directly reflect cellular energy metabolism in living cells, offering ground truth against which computational predictions can be benchmarked. They also boast high sensitivity and reproducibility when using adequate cell numbers and replicates and the ability to test dynamic responses to drug perturbations or substrate changes [57]. Flow cytometry-based methods bring single-cell resolution and multiplexing capacity. SCENITH, for example, can profile multiple metabolic dependencies in parallel across diverse cell subsets in a heterogeneous sample, even ex vivo. These bulk and flow methods are well established, instrumentally accessible, and broadly used in the field, thereby forming a solid experimental anchor for newer computational techniques. However, both Seahorse and flow cytometry-based metabolic profiling readouts still fall short of delivering detailed metabolic information, especially for rare immune cells. In sum, while Seahorse and cytometry-based assays provide valuable kinetic and single-cell insights, they do not directly quantify metabolite pools or pathway-resolved flux, limiting their application on rare immune cells, and require complementary biochemical approaches.

## 4. Emerging Metabolomic Approaches to Rare Cell Populations

Metabolomics consists of the comprehensive and quantitative measurement of small molecules in cells, tissues, and biofluids [69]. Compared to transcriptomics, which reports upstream readouts, metabolomics directly reports pathway intermediates and final products of metabolic reactions and thus is the gold standard for assaying metabolic state [70]. A canonical distinction within experimental designs of metabolomics is between “snapshot” (steady state) and flux tracing. The “snapshot” approach involves rapidly quenching cellular activity to capture instantaneous pool sizes, which identify what metabolites are currently inside a cell and in what quantity. The approach of “snapshot” metabolomics is primarily mass spectrometry (MS) [71] or NMR [72] based and is commonly organized as targeted, which has predefined metabolites with absolute quantification, versus untargeted, which utilizes unbiased, global analysis across thousands of features identified. These two approaches are often combined sequentially to move from discovery to confident annotation and quantification [73,74]. Historically, these technologies demanded substantial material, which often required millions of cells or sizable tissues to reach detection limits (especially for NMR) and to limit technical variability [75]. As a result, classic metabolomics was largely restricted to bulk samples, obscuring heterogeneity in rare immune subsets or small biopsies. Although recent single-cell methods are advancing quickly, the sensitivity, coverage, and quantitation accuracy remain active challenges, reinforcing the dominant role of bulk metabolomics in the field [76,77].

Beyond metabolite-centered readouts, multi-omics approaches leverage the complementarity of metabolite levels and gene expression [78]. By combining transcriptomics and metabolomics, joint pathway analysis tools exist to map metabolite-gene networks that better reflect physiologically relevant readouts than either omics alone. In various cases, pathways or targets undetected by single technology analyses showed up as significant when data are integrated [79,80]. Practically, many researchers now combine extracellular flux phenotyping with ^13^C-tracing and targeted LC-MS to connect macroscopic bioenergetic readouts (OCR/ECAR) to intracellular carbon labeling, offering a coherent pipeline from phenotype to mechanism [81]. In summary, whether as rapid “snapshots” or isotope-resolved flux analysis, metabolomics provides the most direct window into pathway activity. Integrated transcriptome-metabolome frameworks now enable system-level modeling of flux that links molecular regulation to functional metabolism. While sample-size constraints historically limited their application to large cell numbers, continuing advances in sensitivity and single-cell methods are emerging, which offer a chance to overcome these long-standing challenges.

**Figure 1:**
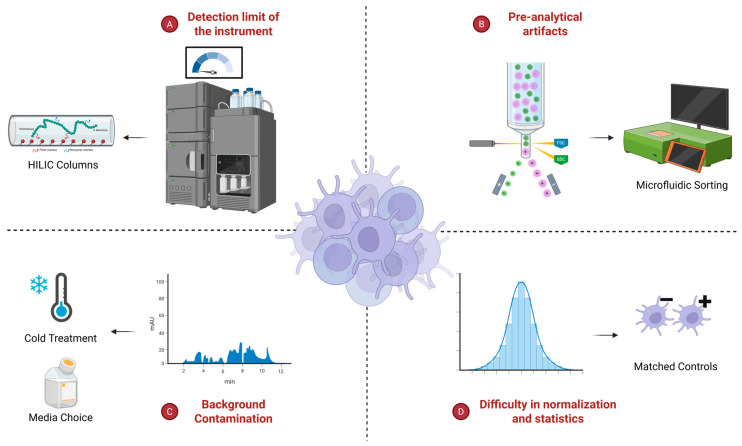
Current challenges of rare cell metabolism investigation. Limited sensitivity is addressed with HILIC to enhance the detection of polar metabolites at low input. Pre-analytical artifacts can be minimized by gentle microfluidic sorting. Background contamination is curtailed via rapid cold handling and defined media. Rigor in normalization/statistics is improved with matched blanks/controls and appropriate denominators (e.g., cell count, protein, DNA).

Studying metabolic states in rare cell types remains technically demanding because metabolite pools are small, dynamic, and easily perturbed by preparation steps (Figure 1). The challenges are: 1. Their low cell numbers push many metabolic intermediates close to the instrument’s limit of detection, amplifying stochastic noise and dropouts. 2. Pre-analytical artifacts can dominate the readout of rare cell types. For instance, traditional fluorescence-activated cell sorting (FACS) induces redox stress during its sorting process, which generates a significant metabolic artifact that overwhelms the physiological metabolic state [82,83]. 3. Background contamination, which can be ignored with high input samples, is now a major source of artifact with low input pipelines, and these metabolites, carried by sheath fluid, plastics, and cell culture media, can obscure the signal of metabolites from rare cells [22,84]. 4. Normalization and statistics are unreliable at the rare-cell limit. Cell numbers, protein numbers, or DNA-based denominators are biased in small numbers, which leads to left-censoring of low-abundance features, which can lead to false discoveries [85,86]. And finally, not for rare cell populations specifically, but loss of physiological context is a central limitation for all in vitro or ex vivo metabolic approaches. Nutrient, oxygen, pH, and cell–cell communication cues in physiologic environments are not recreated in in vitro conditions [87,88,89,90].

Together, these constraints define the measurement problem for rare-cell metabolomics, which is the need for readouts that are both chemically versatile and ultrasensitive while tolerating rigorous blank controls and minimal handling to maximize the physiological relevance of the data generated. Among available platforms, liquid chromatography-mass spectrometry (LC-MS)-based metabolomics has recently been used for the development of multiple novel approaches allowing for both “snapshot” and flux metabolomic measurement on rare cells. Most current high-resolution metabolomic methods combine mass spectrometry with chromatographic separation (LC-MS), which spreads complex mixtures of metabolites in time, allowing for improved sensitivity when using selective mass detection [20]. Historically, LC–MS–based metabolomic methods have typically required 10^5^–10^7^ cells and favored non-polar to mid-polar analytes (e.g., lipids) [21], limiting coverage of highly polar, water-soluble metabolites central to glycolysis, nucleotide, and redox pathways [91,92]. Ongoing improvements in both LC and MS resolution are progressively overcoming these detection blind spots, enabling detailed metabolic analysis in rare cell populations.

One recently developed low-input workflow combined hydrophilic interaction liquid chromatography (HILIC) with a high-resolution Orbitrap MS, enabling detection of ~160 metabolites in less than 10,000 mouse HSCs [22]. Importantly, this study employed multiple approaches to explicitly control background signal and chemical noise. Cell metabolism was immediately quenched following sorting to reduce sorting-induced artifacts by sorting ice-cold cells directly into 80% acetonitrile. Importantly, using this method, metabolite levels in sorted samples closely matched pipetted controls, indicating minimal sorting- or time-induced distortion. Contamination was explicitly audited and suppressed by eliminating sample drying, running sheath-fluid no-cell controls through the metabolomic pipeline, and tuning sorting conditions to limit ion suppression, which increased the number of metabolites above background in low-input samples. Taken together, these design choices improved signal-to-noise at the rare-cell limit and scaled with input, which specifically detects 157 metabolites from 10,000 cells to 222 metabolites from 100,000 cells, providing a practical template for low-input metabolomics with rigorous blanks and stability checks. A complementary targeted protocol further tightened control of sort-related contaminants (matched blanks) and achieved detection of up to ~80 metabolites from as few as 5000 sorted cells, though the true signal and which metabolites it detected depend on the cell type’s size and composition. This method is practical for rare immune subsets from single animals, though by design, it trades coverage for sensitivity and quantitation [84]. These low-input polar methods converge on HILIC as the workhorse for retaining sugar phosphates, nucleotides, and other highly polar metabolites that reversed-phase workflows often bleed through. The improved retention and sharper peaks of HILIC translate to better sensitivity and quantity in small inputs, while the flip side is decreased robustness and reduced reproducibility [93]. Also, since unsaturated lipids are very hydrophobic molecules, they are poorly characterized by HILIC [94].

Aside from LC resolution, MS resolution can also be improved by coupling ultrasensitive ionization and high-resolution analyzers to make single-cell mass spectrometry possible. By integrating single-cell live-cell imaging with single-cell mass spectrometry (SCLIMS), researchers can capture both metabolic profile and oxidative stress in individual cells [23]. In the SCLIMS workflow, live cells are first imaged with a reactive-oxygen-species (ROS) probe to quantify each cell’s oxidative stress, then the same cell is analyzed by MS, producing a metabolite profile that is paired with its oxidative state. Its main advantage lies in the ability to connect metabolic data to observable phenotypes in the same cell. Using this technique, researchers demonstrated strong metabolic heterogeneity across cells with different ROS burdens. Nonetheless, prolonged imaging and the time required for ionization can induce phototoxicity or alter unstable metabolites, potentially introducing artifacts. Another conceptually distinct advance focuses on combining stable-isotope tracing with electrospray-based mass spectrometry (CyESI-MS), which captures real-time pathway activity, allowing researchers to determine which metabolic routes individual cells use [95]. It uses an ultra-fine tip to sample the contents of one cell, then ionizes those molecules with electrospray and measures them in MS. When combined with cells receiving isotopically labeled metabolites, this technique allows for the tracking of individual atoms throughout metabolism [96]. In co-cultures of tumor cells and macrophages, CyESI-MS revealed cell-type-specific metabolic rewiring, in which tumor-associated macrophages show enhanced oxidative phosphorylation and tricarboxylic acid cycle activity, while neighboring tumor cells exhibited elevated glycolysis and nucleotide sugar metabolism. Although this method provides functional insights into metabolic flux at the single-cell level, it demands high analytical resolution, complex data unmixing, and rigorous controls to ensure accurate interpretation of isotope labeling patterns.

Other than increasing the resolution of LC-MS, researchers have also created flow cytometry add-ons to identify rare cell metabolism at single-cell resolution. One of the approaches is known as label-free, high-throughput laser desorption/ionization mass spectrometry (hi-scMet), which combines flow cytometry with nanoparticle-assisted ionization to measure > 100 features per cell across the hematopoietic hierarchy [30]. This technique sorts FASC-identified single cells into nanoparticle-prepared target wells and then uses nanoparticle-assisted MS to read out cells’ small-molecule profile at high throughput. Nanoparticles offer a high surface area for analyte enrichment and produce less background in the low mass-to-charge region in mass spectrometry, which makes them especially valuable for small-molecule metabolite detection. Using this method, the authors identified oxidative pentose phosphate pathway activity as a core regulator of the dormancy-to-active transition of HSCs. Importantly, because the method does not include LC-MS in its system, it is less effective in distinguishing structurally similar metabolites and confirming their identities. In addition, laser-based ionization and fixation procedures can alter redox-sensitive molecules. Thus, hi-scMet offers exceptional throughput and discovery potential on rare cell types but provides limited precision in structural identification and quantification.

Across methods, two major challenges stand out when studying rare cell populations. First, background noise becomes a major limitation when analyzing small numbers of cells. In such cases, it is critical to include matched control samples and carefully monitor contamination to ensure that detected metabolites truly reflect cellular biology rather than signals from buffer solutions or plastic materials [84]. Second, different cell types vary in how well they are represented after sorting and handling. For example, protocols that maintain samples at low temperatures (4 °C) and use HILIC-based chromatography tend to better preserve the metabolic state of quiescent HSCs and many lymphoid cells. However, fragile or granule-rich cells, such as basophils, eosinophils, and tissue macrophages, may be lost or stressed during isolation. Therefore, effective rare cell metabolomics requires matching the platform to biology while enforcing strict matched blank controls to suppress sheath and plastic background so that measured metabolites reflect true cellular chemistry.

## 5. Gaps Between Technical Limitations and Physiological Relevance

One of the challenges with current metabolism is the difference between in vitro and in vivo, especially for immune cells, since their metabolism is exquisitely sensitive to the physiological milieu. This is well illustrated in CD8+ T cells, where physiologically activated cells isolated from secondary lymphoid organs have more oxidative metabolism and channel carbon into anabolic routes such as serine biosynthesis, in sharp contrast to the glycolysis-dominant state observed during in vitro activation [97].

Beyond the in vivo to in vitro divide, the methods used to isolate cells can also alter their metabolic signatures. Conventional fluorescence-activated cell sorting (FACS) employs high-pressure droplet-based sorting, which has been shown to induce metabolic change, including increases in oxidative stress and suppressed mitochondrial metabolism. Sorted cells show altered ratios of GSSG to GSH, NADPH to NADP+, and NAD+ to NADH, with up to a 50% increase in reactive oxygen species observed in cells from FACS relative to unsorted controls [82]. To address this issue and reduce sort-induced stress, microfluidic chip-based sorting has emerged, which reduces the metabolomic changes caused by the droplet-based method [98]. In contrast, droplet sorting produced larger shifts in cellular redox status and pathways linked to transcriptional regulation and mechanical stress signaling. Notably, stress-associated signals were reduced but still detectable in the microfluidic workflow, suggesting that non-mechanical variables during isolation also shape the post-sort metabolic state, e.g., temperature control, media composition, antioxidant capacity, the time out of the incubator, etc.

Temperature control represents another critical factor for preserving native cellular metabolism. Single-cell isolations of immune cells from tissue traditionally require enzymatic incubation at 37 °C. The combination of warm temperature, dissociation stress, and lack of local niche signals may drive cellular stress or adaptive programs, which can result in changes in gene expression and metabolite patterns. Interestingly, a group recently found that use of a psychrophilic protease (a protease with high activation at 6 °C) for digestion better preserves gene expression patterns [99]. Similar considerations apply to metabolomic workflows. Conventional single-cell MS measures live cells over many minutes at room temperature, during which fast-turnover metabolites (ATP/ADP, glycolytic/TCA intermediates, redox couples) drift away from their in vivo baseline. Researchers have mitigated this problem through building a rapid, MS-compatible quench pipeline, which includes volatile salt wash, liquid-nitrogen snap-freeze, vacuum freeze-dry, and freezer storage at −80 °C, which preserves cellular metabolic profile [100].

In parallel with cold workflows, improvements in nanoliter-scale injections have significantly advanced low-input metabolomics [101,102]. Ultra-high-performance liquid chromatography (UHPLC), which uses smaller particles and higher pressures than standard HPLC, can achieve sharper separations and faster run times [103]. When coupled with nano- or capillary-flow systems, UHPLC allows for true nanoliter loop injections, concentrating analyte on columns, reducing dispersion, and increasing sensitivity for rare cell samples. Modern systems (e.g., Vanquish Neo, 1 nL–100 µL/min; ≤1500 bar [104]) demonstrate excellent precision and expanded dynamic range under these conditions. Together, these innovations form an integrated pipeline that connects physiological isolation with high-sensitivity metabolic measurement. This end-to-end approach brings the field closer to capturing the true metabolic state of rare immune cells in their native context.

## 6. Unveiling the Spatial Landscape of Cellular Metabolism

Though LC-MS-based metabolomics offers deep chemical coverage, it homogenizes tissue and therefore loses spatial context, which makes it hard to read out metabolic states in niches or capture metabolite exchange between neighboring cells. Spatial transcriptomics are emerging nowadays because they preserve cellular neighborhood information while delivering transcriptomes at single-cell resolution in intact tissue. Platforms like Slide-seq and Visium have demonstrated high-resolution maps of gene expression that situate cell states within their native microenvironments [16,17]. These spatial maps provide a platform for researchers to map metabolism.

Mass spectrometry imaging (MSI), as a complementary technology to slide-seq, also provides a foundation to map metabolites directly onto tissue architecture. By scanning a tissue section point-by-point and recording a full mass spectrum at each point, MSI draws a map of metabolites across the tissue to infer local pathway activity and potential metabolite exchange [24]. Recent advances in higher spatial resolution modalities and post-ionization strategies are increasing MSI’s sensitivity and pushing toward subcellular scales, making MSI increasingly suited for precise metabolic readouts in rare cell types [25,26]. The matrix-assisted laser desorption ionization (MALDI) imaging mass spectrometry can detect metabolites in 250 pmol amounts, including endogenous metabolites (peptides, lipids, hormones) and exogenous metabolites (drugs and drug-related metabolites, tracers, toxins) [105]. Sun et al. have found pyrrolin-5-carboxylate reductase 2 (PYCR2) and uridine phosphorylase 1 (UPase1) were altered in esophageal cancer patients using high-resolution MSI [106]. In practice, careful choice and optimization of ionization methods remain important because they shape which metabolite classes are detected [24,107]. Consequently, MSI is becoming a practical backbone, enabling metabolic signals to be localized to precise tissue niches in disease contexts.

## 7. From Correlation to Causation: CRISPR Screening Closes the Loop

The technologies surveyed above are powerful for charting metabolic states, but fundamentally can only reveal correlative associations. Establishing causal relationships requires perturbing genes and observing phenotypic consequences in relevant contexts and is critical to defining the metabolic signaling and processes that shape cellular function. The continued development of CRISPR pooled screening via genome-scale knockout (CRISPR KO), repression (CRISPRi), and activation (CRISPRa) enables systematic testing of whether a gene is required or sufficient to drive a metabolic phenotype, combining high throughput with causal inference. Landmark implementations now place these screens directly in vivo, revealing dependencies invisible in dish culture [31]. For example, in melanoma models under immune pressure, pooled in vivo KO screens uncovered immune evasion nodes (e.g., PTPN2) that modulate response to checkpoint blockades, highlighting microenvironment-restricted metabolic and signaling liabilities [32]. Beyond tumor cells, pooled CRISPR screening has increasingly been applied to primary immune cells. In human T cells, genome-wide CRISPR KO established tractable screening despite low lentiviral transduction and limited ex vivo lifespan, defining regulators of stimulation and suppression with direct relevance to immunotherapy [33]. Similar concepts extend to more upstream antigen-presenting cells (e.g., dendritic cells), where CRISPR screens dissected TLR4-TNF regulation [34]. Together, these advances move screening beyond cancer cell lines to immune regulators and reveal upstream targets.

To read out metabolic causality at scale, several groups have built metabolism-focused CRISPR libraries that densely cover enzymes, transporters, and cofactors. The Birsoy group has built both mouse and human CRISPRa libraries, which target 2989 metabolic genes [108], and CRISPR KO libraries, including 2981 metabolic genes [109], enabling bidirectional screens across central carbon, lipid, and amino acid metabolism. The CRISPRa library is critical for testing sufficiency, revealing redundant pathways, and defining rate-limiting steps that knockout screens can miss. The human CRISPR libraries are especially important for immunometabolism because they operate directly in human cells and models, improving translational relevance for drug targets in central carbon, lipid, and amino-acid metabolism, and allowing cross-species validation against mouse libraries. Together, these metabolism-centric CRISPR libraries provide a scalable framework to turn correlative metabolic signatures into causal genetic pathway maps across immune contexts, and the repertoire is rapidly expanding as new pathway-focused and commercial libraries continue to appear.

CRISPR screen readouts now expanded beyond enrichment/depletion to include high-content molecular phenotypes. Perturb-seq links pooled CRISPR perturbations to scRNA-seq, resolving on-target programs, compensatory circuits, and context-specific interactions [35]. Paired platforms such as CROP-seq and ECCITE-seq further tag guides and add surface-protein and TCR clonotype modalities [110,111]. A genome-scale Perturb-seq study in 2022 profiled thousands of perturbations and catalyzed community resources that standardize datasets and support benchmarking and reuse [112]. For metabolism, these single-cell readouts enable mapping of how gene perturbations shift pathway signatures (e.g., glycolysis, fatty-acid oxidation, one-carbon metabolism) and ranking of causal nodes.

This approach is especially valuable for rare cell metabolism. Pooled libraries allow for testing thousands of perturbations within a single mixed culture or in vivo niche after pre-enrichment, and single-cell lineage assigns each rare cell both its transcriptome and its guide identity, which turns genetic instructions into transcriptional programs with cell-type resolution. CRISPRi/CRISPRa are especially attractive because they avoid double-strand breaks that can compromise fragile or quiescent populations, and their graded knockdown/activation process helps reveal rate-limiting steps rather than binary lethality. Coupling pooled CRISPR screens with single-cell profiling (e.g., Perturb-seq/CROP-seq/ECCITE-seq) maps rare cell transcriptomes with their guide identities, making causal identifications from genetic instructions to transcriptions. The emergence of accessible metabolic libraries further increases the accessibility of CRISPR screens.

**Figure 2:**
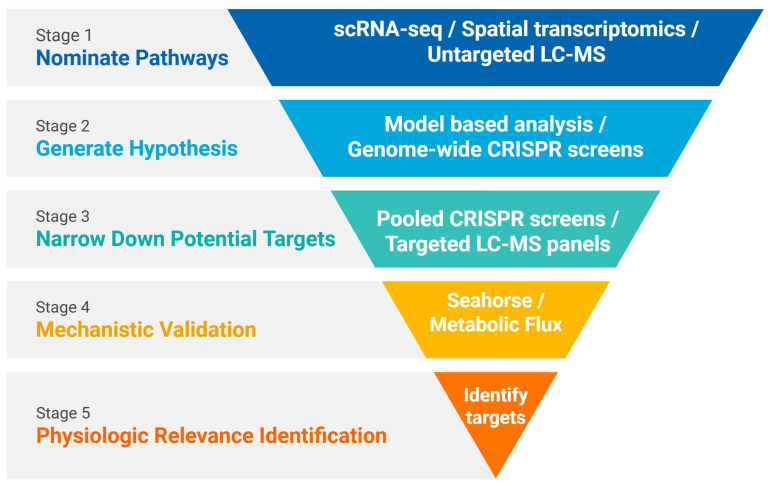
Causality pipeline for immunometabolism. Targets are narrowed through coherent metabolic approaches.

Practical constraints remain important for CRISPR screens, particularly for scarce populations. Classical pooled designs target low multiplicity of infection (MOI) (0.3–0.5) to ensure one perturbation per cell and require high coverage (commonly ~200–500 per sgRNA) for robust statistics [36]. For genome-scale libraries (50–100 k guides), total input can easily climb to tens of millions of cells, which is not feasible for rare cells and can bias towards expandable clones or distort state distributions. Together, these factors explain why “big inputs” are problematic for rare cells and motivate compact, pathway-focused libraries and improved delivery. More research is now developing a more precise CRISPR screen that has high infection efficiency and can be performed in low cell amounts. Emerging high-efficiency, low-input CRISPR screening strategies will enable these perturbation studies to be incorporated into a streamlined causality pipeline (Figure 2). 

## 8. Conclusions

Integrated, causality-focused pipelines can move immunometabolism from descriptive atlases to mechanistic maps (Figure 2). Stage 1 (Nominate pathways): discovery begins with scRNA-seq and spatial transcriptomics, now complemented by untargeted LC-MS, to nominate candidate pathways and putative cell–cell metabolite exchange within intact tissue. Stage 2 (Generate hypothesis): model-based analyses (e.g., GEMs, Compass) convert these profiles into flux-constrained pathway hypotheses, while pooled CRISPR screens in parallel highlight genes whose perturbation shifts these states. Stage 3 (Narrow down targets): hits are refined using confirmatory pooled CRISPR and targeted LC-MS panels that resolve pathway-level changes and prioritize tractable nodes. Stage 4 (Mechanistic Validation): quantitative validation couples metabolic flux analysis with population-level Seahorse assays (OCR/ECAR) and, when needed, single-cell energetic profiling (e.g., SCENITH). Targeted CRISPR screens then test the necessity and sufficiency of candidate enzymes and transporters in the relevant context. Stage 5 (Druggable target identification): functional readouts including effector function, cytotoxicity, cytokines, and in vivo control to nominate druggable targets. Throughout, the CRISPR “right-rail” can plug into multiple stages, including nomination, hypothesis generation, and quantification, to iteratively connect gene perturbations to pathway function across scales.

For rare cell populations, two pragmatic entry points are (1) low-input LC-MS workflows to capture polar metabolites and generate pathway leads or (2) metabolism-focused CRISPR libraries to screen enzymes, transporters, and cofactors when material limits preclude comprehensive metabolite profiling. Subsequent mouse knockout or knockin models provide orthogonal, in vivo causal validation under physiological nutrient, oxygen, and immune constraints. Metabolic effects that recur across methods (e.g., appear in both scRNA-seq modeling, CRISPR screen, and LC-MS) warrant elevated confidence and prioritization for mechanistic workup. Importantly, this pipeline is not restricted to rare populations and can be readily applied to more abundant cell types as well. Looking forward, tighter coupling of CRISPR screens with chemical flux reporters and single-cell fluxomics will be the future method for researchers to find physiologically relevant targets more easily. With these pieces in place, immunometabolism can move from descriptive atlases to causal maps of all pathways, revealing druggable nodes that reshape immune function precisely where it matters: in the tissue, in the moment, and in the rare cells that tip the balance.

## Figures and Tables

**Table 1:** Summary of current tools for cellular metabolic analysis and their applicability to rare cell populations.

Method	Extended Approaches	Cell Input Requirement	Throughput	Rare Cell Type Suitability	References
scRNA-seq	Computational scRNA-seq prediction (e.g., Compass)/Spatial transcriptomics (e.g., slide-seq)	500 sequenced cells from specific population	High	Good	[13,14,15,16,17]
Extracellular flux analysis	scFBA	Depends on cell types 1 × 10^4^ tumor cells/well 1 × 10^5^ immune cells/well	Low	Low	[18,19]
MS	HILIC-MS/SCLIMS/MSI for spatial information	Conventional bulk LC-MS: 1 × 10^5^~1 × 10^7^ cells/sample Optimized targeted methods: 1 × 10^4^	Moderate-High	Bulk LC-MS: Low MSI/HILIC-MS: Good	[20,21,22,23,24,25,26]
Flow cytometry	Spectral flow cytometry-based panels (e.g., SCENITH, CENCAT)/hi-scMet	as low as 500 cells, depends on panel	High	Good	[27,28,29,30]
CRISPR screen	Perturb-seq	1 × 10^6^ cells	Very high	Good	[31,32,33,34,35,36]

## Data Availability

The original contributions presented in this study are included in the article. Further inquiries can be directed to the corresponding author.

## Abbreviations

The following abbreviations are used in this manuscript:

ATP	Adenosine triphosphate
BiGG	Biochemically, Genetically and Genomically structured models database
BMI	Body mass index
CCC	Cell–cell communication
CENCAT	Cellular energetics through noncanonical amino acid tagging
CRISPR	Clustered Regularly Interspaced Short Palindromic Repeats
CRISPRa	CRISPR activation
CRISPRi	CRISPR interference
CyESI-MS	Cytoplasmic electrospray ionization mass spectrometry
ECAR	Extracellular acidification rate
FACS	Fluorescence-activated cell sorting
FBA	Flux Balance Analysis
GEMs	Genome-scale metabolic models
GSH	Reduced glutathione
GSSG	Oxidized glutathione
HCA	Human Cell Atlas
hi-scMet	High-throughput single-cell metabolomics
HILIC	Hydrophilic interaction liquid chromatography
HSCs	Hematopoietic stem cells
KO	Knockout
LC-MS	Liquid chromatography–mass spectrometry
MEMs	Model Extraction Methods
mCCC	Metabolite-mediated cell–cell communication
MOI	Multiplicity of infection
MS	Mass spectrometry
MSI	Mass spectrometry imaging
NAD+	Nicotinamide adenine dinucleotide (oxidized)
NADH	Nicotinamide adenine dinucleotide (reduced)
NADP+	Nicotinamide adenine dinucleotide phosphate (oxidized)
NADPH	Nicotinamide adenine dinucleotide phosphate (reduced)
NMR	Nuclear magnetic resonance
OCR	Oxygen consumption rate
ROS	Reactive oxygen species
SCLIMS	Single-cell live-cell imaging–mass spectrometry
SCENITH	Single-cell energetic metabolism by profiling translation inhibition
scFBA	Single-cell Flux Balance Analysis
scRNA-seq	Single-cell RNA sequencing
sgRNA	Single-guide RNA
TCA	Tricarboxylic acid cycle
TCR	T cell receptor
Th17	T helper 17 cell
TLR4	Toll-like receptor 4
TNF	Tumor necrosis factor
Treg	Regulatory T cell
UHPLC	Ultra-high-performance liquid chromatography
XF	Extracellular flux
